# Biodegradability Assessment of Polyester Copolymers Based on Poly(ethylene adipate) and Poly(ε-caprolactone)

**DOI:** 10.3390/polym14183736

**Published:** 2022-09-07

**Authors:** Leonard Ionut Atanase, Slim Salhi, Oana Cucoveica, Marijana Ponjavic, Jasmina Nikodinovic-Runic, Christelle Delaite

**Affiliations:** 1 Faculty of Medical Dentistry, Apollonia University of Iasi, 700511 Iasi, Romania; 2 Academy of Romanian Scientists, 050045 Bucharest, Romania; 3 Laboratoire de Chimie Appliquée, Faculté des Sciences de Sfax, University of Sfax, Sfax 3029, Tunisia; 4 “Cristofor Simionescu” Faculty of Chemical Engineering and Environmental Protection, “Gheorghe Asachi” Technical University of Iasi, 700050 Iasi, Romania; 5 Department of Electrochemistry, Institute of Chemistry, Technology and Metallurgy, University of Belgrade, 11000 Belgrade, Serbia; 6 Institute of Molecular Genetics and Genetic Engineering, University of Belgrade, 11000 Belgrade, Serbia; 7 Laboratoire de Photochimie et d’Ingenierie Macromoleculaires (LPIM), University of Haute Alsace, 68100 Mulhouse, France

**Keywords:** PCL, PEA, polyesters, copolymers, biodegradability

## Abstract

Biodegradable polymers contain chains that are hydrolytically or enzymatically cleaved, resulting in soluble degradation products. Biodegradability is particularly desired in biomedical applications, in which degradation of the polymer ensures clearance from the body and eliminates the need for retrieval or explant. In this study, a homologues series of poly(ε-caprolactone)-b-poly(ethylene adipate)-b-poly(ε-caprolactone) (PCL-b-PEA-b-PCL) block copolymers, with constant PEA molar mass and different PCL sequence lengths was obtained. The starting point of these copolymers was a dihydroxy-PEA precursor with a molar mass (M_n_) of 2500 g/mol. Mn values of the PCL varied between 1000 and 10,000 g/mol. Both the precursors and the copolymers were characterized using different physicochemical methods, such as: NMR, SEC, Maldi-TOFF, DSC, and ATG. The molecular characteristics of the copolymers were in a direct correlation with the sequence length of the PCL. Enzymatic degradability studies were also conducted by using cell-free extract containing *Pseudomonas aeruginosa* PAO1 for 10 and 21 days, and it appeared that the presence of the PEA central sequence has an important influence on the biodegradability of the copolymer samples. In fact, copolymer PCL_7000_-PEA_2500_-PCL_7000_ had a weight loss of around 50% after 10 days whereas the weight loss of the homopolymer PCL, with a similar M_n_ of 14,000 g/mol, was only 6%. The results obtained in this study indicate that these copolymer samples can be further used for the preparation of drug delivery systems with modulated biodegradability.

## 1. Introduction

For the last decades, a wave of interest in the synthesis of biodegradable polymers has been developed by different research groups [1,2,3,4]. During the sixties, the first applications of these polymers appeared. Bringing an ecological response to replace non-biodegradable plastics, biodegradable polymers are also of great importance for the biomedical and pharmaceutical fields due to their low cost, biocompatibility, flexibility, and minimal side effects. The biocompatibility, low toxicity and chemical functionalities of these polymers make them suitable for a variety of biomedical applications that include nanocarriers for the delivery of bioactive molecules, in tissue engineering and scaffolds, cardiology, orthopedics, etc. [5,6].

Controlled drug delivery has become one of the important research areas in polymeric biomaterials during the past few years [7,8,9,10]. A drug delivery system (DDS) is defined as a formulation or a device that enables the introduction of a therapeutic substance into the body and improves its efficacy by controlling the rate, time, and place of release [11].

Drug-loaded nanoparticles (NPs), generally based on biocompatible and biodegradable polymers, are characterized by high drug encapsulation efficiency; improved drug bioavailability; solubility and retention time; enhanced chemical and biological stability; controlled drug release rate; and a wide variety of administration routes [12,13].

Polymers suitable for the preparation of DDS must meet several requirements, such as biodegradability, biocompatibility, suitable mechanical properties, and easiness of handling and processing [14]. Aliphatic polyesters, such as poly(lactic acid) (PLA), poly(glycolic acid) (PGA), poly(lactic-co-glycolic acid) (PLGA), and poly(ε-caprolactone) (PCL), are extensively used in biomedical applications due to their very remarkable characteristics: biodegradability, biocompatibility, and mechanical properties. Moreover, it has been found that the physical properties of such polyesters (molecular weight, melting point, or degree of crystallinity) affect the drug release behavior [15,16,17,18].

Among these polyesters, PCL, which is usually produced by ring-opening polymerization of ε-caprolactone [19,20,21], is a fully biodegradable semi-crystalline FDA-approved polymer that has a low melting point (T_m_ = 60 °C) and a low glass transition temperature (T_g_ = −60 °C) [19,22]. The preparation of NPs out of pure PCL, with diameters from 190 to 350 nm, has only been investigated in a few studies [23,24,25,26].

However, the high degree of crystallinity of PCL limits its biodegradation rate and thus, it is necessary to copolymerize or mix the PCL homopolymer with homopolymers that biodegrade faster [27]. Such biodegradable polymers are able to undergo chemical bond scission and are accompanied by physical erosion when exposed to the biological environment. In fact, molecular chain scission can be initiated passively by hydrolysis, or actively by enzyme-catalyzed hydrolysis [20]. Degradation corresponds to the formation of polymers with lower molecular weights, due to the cleavage of chemical bonds. After a sufficient number of scissions, the chains are reduced to the state of monomers or small soluble oligomers, and can therefore be evacuated, which leads to a loss of weight of the material. Throughout the degradation of polymers, their physical and chemical properties will change, such as crystallinity, molar mass, pH, and mechanical properties.

Poly(ethylene adipate) (PEA) is a novel biodegradable aliphatic polyester, which has gained increasing interest due to its low crystallinity. Moreover, this type of polymer degrades faster than most polyesters used as DDS, including PCL [27,28] and thus, copolymers based on PCL and PEA could be of high interest. It is known that, in many cases, homopolymers do not present the “optimal” set of properties for practical uses. Therefore, copolymers have become more and more important, since their characteristics can be easily tailored to fit specific applications whereas their physical properties are strongly affected by the molecular composition, molar mass, structural arrangement of molecular units, sample crystallinity, etc.

The aim of the present study was to synthesize, for the first time, a homologues series of new poly(ε-caprolactone)-b-poly(ethylene adipate)-b-poly(ε-caprolactone) (PCL-b-PEA-b-PCL) block copolymers, with constant PEA molar mass and different PCL sequence lengths, by melt polycondensation technique of ε-CL starting from a dihydroxy-PEA. After the full physicochemical characterization of the copolymers, their enzymatic biodegradability was assessed.

## 2. Materials and Methods

### 2.1. Materials

Adipic acid (>99%), succinic acid (>99%), dodecanedioic acid (>99%), dimethyl adipate (>99%), titanium butoxide (>99%), ethylene glycol (>99.8), PCL homopolymers with molar masses of 14,000 and 80,000 g/mol were purchased from Sigma Aldrich (Steinheim, Germany). All the other products and reagents were used without further purification.

### 2.2. Methods

#### 2.2.1. Synthesis of PEA Precursors (PEA-diOH)

**Scheme 1:**
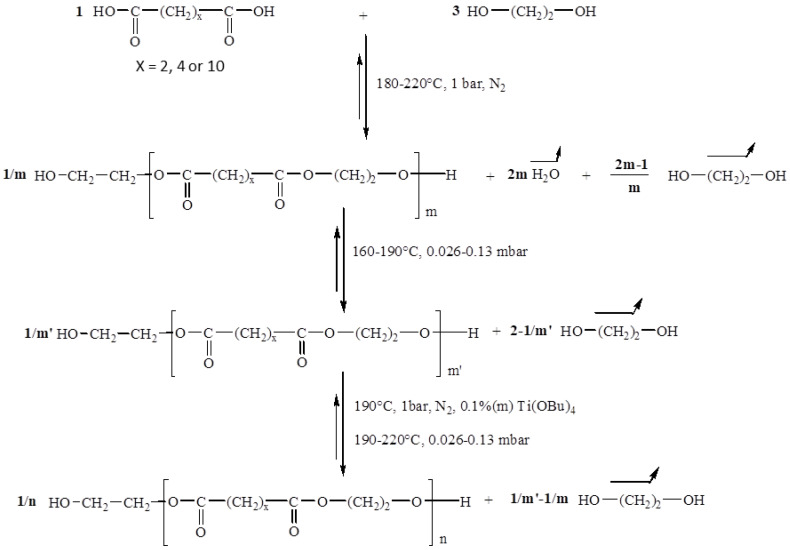
Reaction mechanism of the synthesis of α,ω-dihydroxy aliphatic oligoesters.

PEA-diOH was synthesized by mass polycondensation between ethylene glycol and adipic acid with an initial molar ratio [OH]_0_/[COOH]_0_ equal to 3. The reaction was carried out in three stages, as illustrated in Scheme 1.

The first step, carried out under atmospheric pressure and at a temperature ranging from 180 to 220 °C, corresponds to the esterification of the diacid which reacts with ethylene glycol. The second stage, carried out at 190 °C under reduced pressure, eliminates the excess diol introduced at the beginning of the reaction. Polymer synthesis takes place in the last step after the addition of a transesterification catalyst. By varying the duration of the poly(transesterification) step, polyesters of different molar mass can be obtained. The experimental parameters used in this study for the synthesis of the PEA precursors are provided in Table 1. Increasing the duration of this synthesis phase, at constant pressure, provides access to high molecular weight polymers.

#### 2.2.2. Synthesis of PCL-b-PEA-b-PCL Block Copolymers

**Scheme 2:**
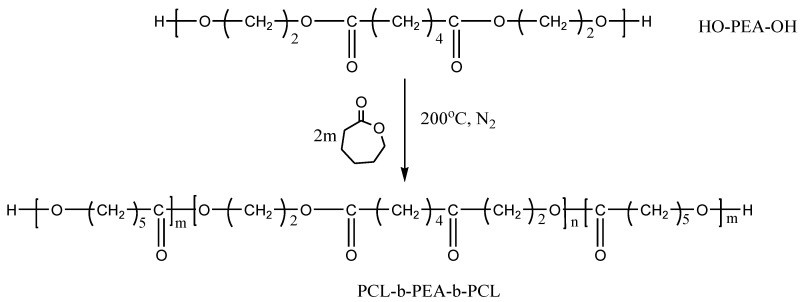
Reaction scheme for the synthesis of PCL-b-PEA-b-PCL copolymers.

The polymerization of ε-caprolactone (ε-CL) starting from the PEA-diOH precursor was carried out by the technique of high-temperature bulk polycondensation, as illustrated in Scheme 2. In a typical reaction, 1 g of PEA and 1 g of ε-CL were introduced into a 50 cm^3^ reactor fitted with a central mechanical anchor stirrer, a nitrogen inlet, and an outlet. The mixture was brought to 180–210 °C. Once it was melted and homogeneous (5 min), 0.1 wt.% of a transesterification catalyst (i.e., 155 μL of a 1% solution in dichloromethane) is added using a hypodermic syringe. The reaction was continued for 30 min. The sample was analyzed after cooling without further purification.

#### 2.2.3. Biodegradability Studies

(a) Preparation of cell-free extract total protein extract for degradation experiments

*Pseudomonas aeruginosa* PAO1 (ATCC 15692) was grown in LB medium [29] with the addition of olive oil (0.1%, *v*/*v*) and PCL powder (1 g/L) at 30 °C for 48 h and 180 rpm. Cells were harvested by centrifugation (Sorvall Centrifuge, DuPont Instruments, Wilmington, DE, USA) at 5000 rpm and 4 °C, and supernatant and cells were separated. The cell pellet was resuspended in the 20 mM phosphate buffer pH 7.4 (10 mL), disrupted by sonication applying 4 short bursts for 20 s at 25 kHz with 20 s cooling in between (Soniprep 150, MSE, London, UK), and centrifuged for 30 min at 14,000 rpm at 4 °C (Eppendorf Centrifuge 5417 R, Hamburg, Germany). This cell-free extract was mixed with the culture supernatant (1:1 ratio) in order to obtain the complete intra- and extracellular protein extract. Protein concentration was determined using a Bicinchoninic Acid Kit for Protein Determination (Sigma-Aldrich, Munich, Germany).

(b) Enzymatic degradation in liquid medium

The enzymatic degradation experiments were performed in 100 mL flasks where dialysis bags with approximately 100 mg of each sample were placed into 50 mL of phosphate buffer solution (pH 7.4). Polymer samples in the form of powder were put in dialysis bags where 2 mL of *Pseudomonas aeruginosa* PAO1 cell-free total protein extract was added. The degradation procedure was adopted from Ponjavic et al. [30,31] with some adaptations. The samples were incubated at 37 °C for 10 days and 21 days. In the batch that was incubated for 21 days after a degradation period of 10 days, cell-free extract within the dialysis bag was exchanged with the fresh aliquot. Two replicates were removed out of the buffer solution at predetermined time intervals (10 and 21 days), washed with distilled water, and dried at room temperature till the constant weight was reached.

The degradation rate was estimated by determining the weight loss of the polymer samples by comparing weight after degradation of dry sample (*m*_1_) at a specific time with the starting weight (*m*_0_) according to the equation:

$$weight\;loss\;\left(\%\right)=\frac{m_{0}-m_{1}}{m_{0}}\times 100$$

#### 2.2.4. Characterization Techniques

^1^H and ^13^C NMR spectra were acquired on a Bruker AC-400F operating at 400 MHz in CDCl_3_-d6 at room temperature. The reference used to measure the chemical shifts is deuterated chloroform (CDCl_3_) (δ = 7.26 ppm in ^1^H NMR and δ = 77 ppm in ^13^C NMR). For the ^13^C NMR spectra, the Inverse Gated sequence is used (the decoupler is on only during the FID acquisition and is stopped in the relaxation interval which precedes the next pulse with a D1 delay of 2 s) where the NOE effect (Nuclear Overhauser Effect) is removed. In order to obtain quantitative spectra, a small quantity of chromium acetylacetonate Cr(C_5_H_7_O_2_)_3_, a paramagnetic compound, is added to the samples to be analyzed, in order to homogenize and accelerate the relaxation of the carbons.

The SEC analyzes were performed on the following system: WATERS-type 510 HPLC pump/Rheodyne-type injector/detector: differential refractometer (WATERS RI-410)/HPLC tetrahydrofuran eluent at a flow rate of 0.5 mL·min^−1^/60 cm PL-gel^®^ type columns having porosities of 500, 100, and 50 Å and having a particle size of 5 μm/injection of 100 μL of a 1 wt.% solution. Computer signal processing was performed using PL Caliber software (Polymer Laboratories, Church Stretton, UK). Molar masses were determined using polystyrene and MALDI-TOF MS calibration curves.

The MALDI-TOF MS analyses (matrix-assisted laser desorption/ionization coupled to a time-of-flight mass spectrometer) were carried out on a Voyager Elite time-of-flight mass spectrometer (PerSeptive Biosystems, Framingum, MA, USA) equipped with a device extraction delay and a collinear electrostatic reflector. The spectra presented in this study were recorded in positive mode using the electrostatic reflector and delayed extraction. They correspond to the sum of 256 consecutive laser shots at a frequency of 2 Hz. The mass calibration was carried out using the times of flight of ions observed for compounds of known mass (peptide). The spectrometer is equipped with a laser source (VSL 337 ND Laser Science Inc., Newton, MA, USA) whose characteristics are as follows: λ = 337 nm, pulse: 3 ns, fluence: 200 mJ/pulse. The matrices used are 2,5-dihydroxybenzoic acid (DHB) and alpha-cyano-4-hydroxycinnamic acid (HCCA) dissolved in THF (10 mg.mL^−1^). The product to be analyzed is dissolved in THF (1 mg.mL^−1^). A volume of 10 μL of this latter solution is mixed with 100 μL of the solution containing the organic matrix and then 1 μL of the mixture is deposited on a metal target (5 mm in diameter). The sample is analyzed after evaporation of the THF.

Differential scanning calorimetry (DSC) analyses of the polymers were carried out on a TA-Instrument DSC 2920 Modulated device under nitrogen flushing and equipped with an LNCA type cooling system (cooling with liquid nitrogen). An amount of 2 to 5 mg of the polymer was used for the analyses. The temperature rise and fall rates are 20 °C/min. The glass transition temperatures (T_g_) correspond to the inflection point, and the melting temperatures (T_m_) correspond to the minimum of the endotherm.

Thermogravimetric analyses (TGA) were carried out on a TA-Instrument Q50 device, under nitrogen sweeping. The samples to be analyzed (about 10 mg) are placed in a platinum boat. The temperature rise rate was 20 °C/min.

ATR-Fourier Transform Infrared Spectroscopy (ATR-FTIR) of PCL-b-PEA-b-PCL copolymer samples before and after a predetermined time of degradation were recorded using an IR-Affinity spectrophotometer (NICOLET iS10, Thermo Scientific, Waltham, MA, USA). The number of scans was fixed to 32 with a resolution of 4 cm^−1^ at room temperature. All the scans were carried out within the same predefined range of 4000 to 400 cm^−1^.

## 3. Results

### 3.1. Synthesis and Characterization of PEA Precursors

**Figure 1:**
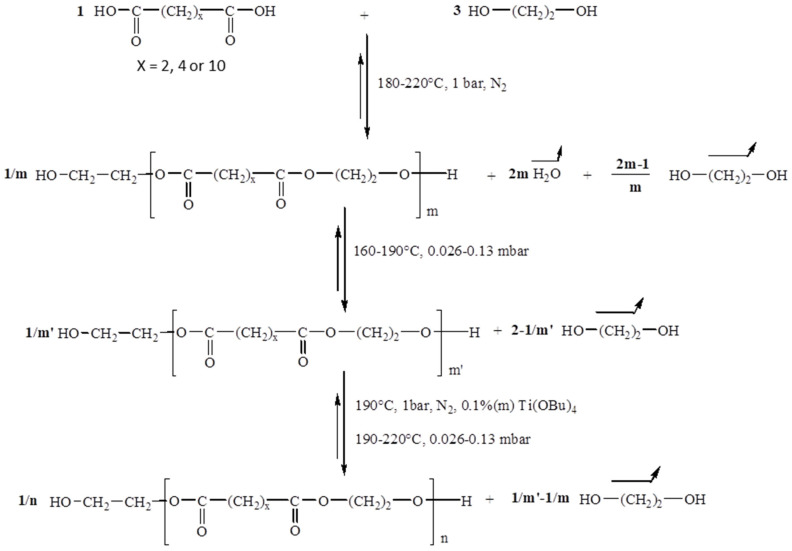
^1^H NMR spectrum of PEA-2 sample in CDCl_3_. (* satellite peaks).

The ^1^H NMR spectrum and peak assignments for sample PEA-2 are given in Figure 1. One can note the presence of methylene protons at α and β of the terminal hydroxy function respectively at 3.81 ppm (H4) and 4.20 ppm (H5); at 3.69 ppm, the triplet related to the methylene group in α of the ether functional group of the 2,2′-oxydiethylene (DEG; H6) type entities which are formed following etherification reactions between two linear chains with hydroxy ends; at 3.72 ppm the singlet corresponding to residual synthetic ethylene glycol (H9); at 4.31 ppm the corresponding very fine peak was attributed to the methylene protons (H8) of the cyclic tetramer of PEA (C2; 2 repetition units ether unit).

**Figure 2:**
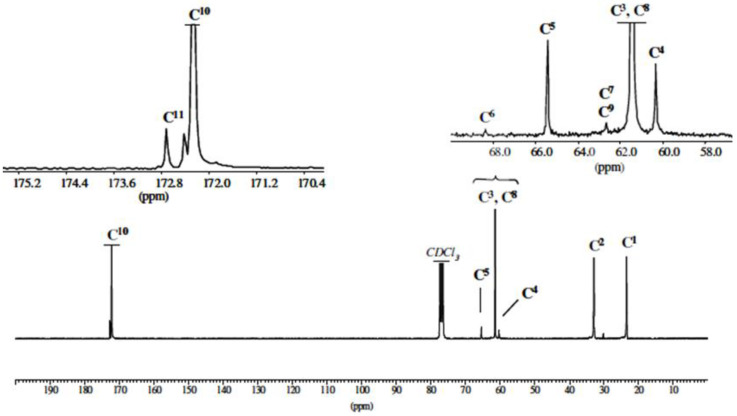
^13^C NMR spectrum of PEA-2 sample in CDCl_3_.

The ^13^C NMR study confirmed the structure of the synthesized polymer (Figure 2).

In particular, the zoom of the 56–69 ppm region shows the presence of low-intensity signals at 60.42 and 65.50 ppm characteristic of the methylene carbons, respectively at α (C4) and β (C5) of the terminal hydroxy function. The peak at 172.72 ppm can be attributed to the specific C11 carbonyl of the cyclic tetramer.

**Figure 3:**
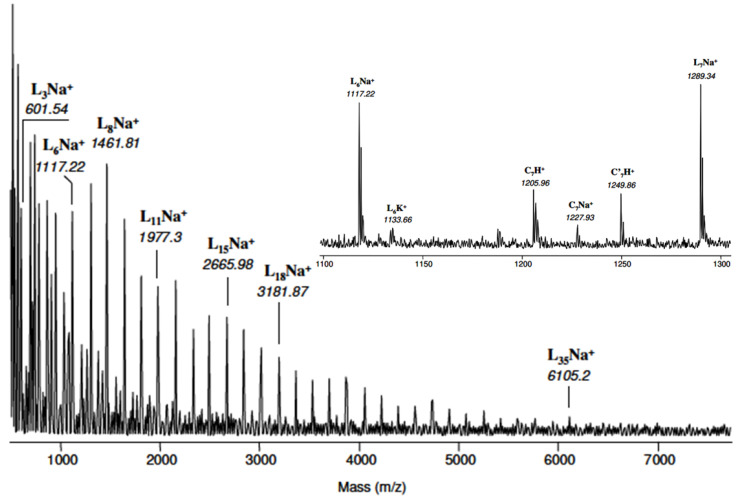
MALDI-TOF chromatogram of PEA-2 sample.

The MALDI-TOF mass spectrum of PEA-2 is represented in Figure 3. It can be observed that the presence of a series of high-intensity peaks separated by a mass of 172 Th corresponds to the molar mass of the PEA repeat pattern. This series was attributed to linear α,ω-dihydroxy oligomers ionized by Na^+^ and denoted LnNa^+^ (*n* = 3 to 35). A finer analysis of the mass spectrum can be carried out in the 1000–2000 Th region. For example, the specific spectrum of a 1100–1300 Th region indicates the existence, in addition to 1117.22 and 1289.34 Th (L6Na^+^ and L7Na^+^), of four low-intensity massifs which can be attributed as follows: (i) 1133.66 Th to linear species L6 ionized by K^+^ and denoted L6K^+^; (ii) 1205.96 and (iii) 1277.93 Th to cyclic macromolecules comprising 7 repeating units protonated and ionized by Na^+^ (denoted respectively C7H^+^ and C7Na^+^); (iv) at 1249.86 Th to cyclic macromolecules with 7 units and containing a 2,2′-oxydiethylene unit and protonated (denoted C’7H^+^).

In view of these results, one can note the greater sensitivity of cyclic species to protonation during MALDI analysis compared to linear macromolecules which are detected only in their forms ionized by Na^+^ and K^+^. Moreover, it was noticed that only the cyclic species contain a DEG (C’n) type ether unit in their structure, unlike the linear macromolecules in which the ether units are not present.

**Figure 4:**
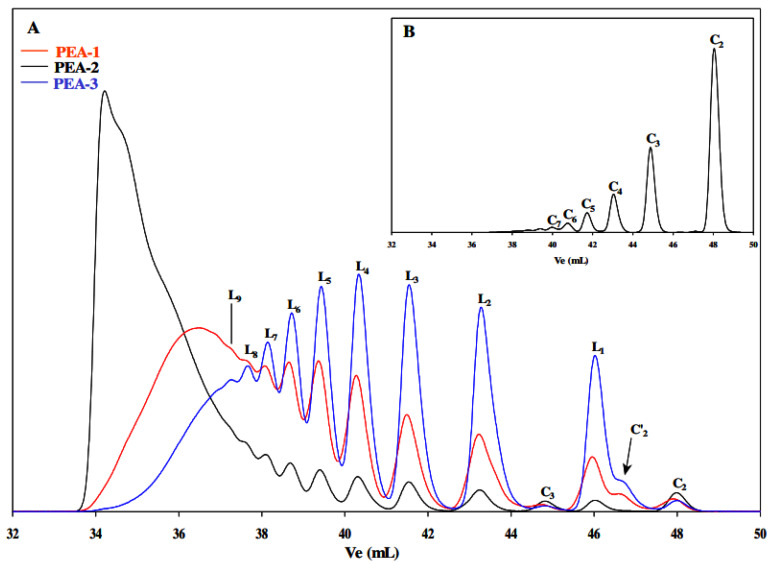
SEC chromatograms of (**A**): PEA-1, PEA-2, and PEA-3: identification of linear dihydroxy macromolecules (Ln), cyclic oligomers with and without DEG motif (C’n and Cn respectively); (**B**) sample obtained after the cyclodepolymerization of PEA-2.

The SEC chromatograms of the three synthesized PEA polyesters are shown in Figure 4. The high-intensity peak present in the region of high elution volumes is specific for the linear oligomer L1 with a degree of polymerization (DP) equal to 3. The low-intensity peaks and shoulder detected in the elution volume zone between 44 and 49 mL were attributed, respectively, to the cyclic esters C3, C′2, and C2, and this by comparison with the SEC chromatogram characteristic of the cyclic species Cn (*n* from 2 to 7) obtained after the cyclodepolymerization of PEA-2.

The molecular characteristics of the PEA precursors synthesized in this study are given in Table 2.

In this step, a series of di-hydroxylated PEAs were obtained and characterized from a physicochemical point of view. The obtained results demonstrated that the PEA-2 sample has the optimal characteristics for further synthesis of PCL-b-PEA-b-PCL block copolymers.

### 3.2. Synthesis and Characterization of Block Copolymers

The polymerization of ε-CL starting from the PEA-2-diOH sample was carried out by the mass polycondensation technique at high temperatures. By varying the amount of the monomer, a series of copolymers was obtained with the same length of the PEA central sequence, but different molar masses for PCL.

**Figure 5:**
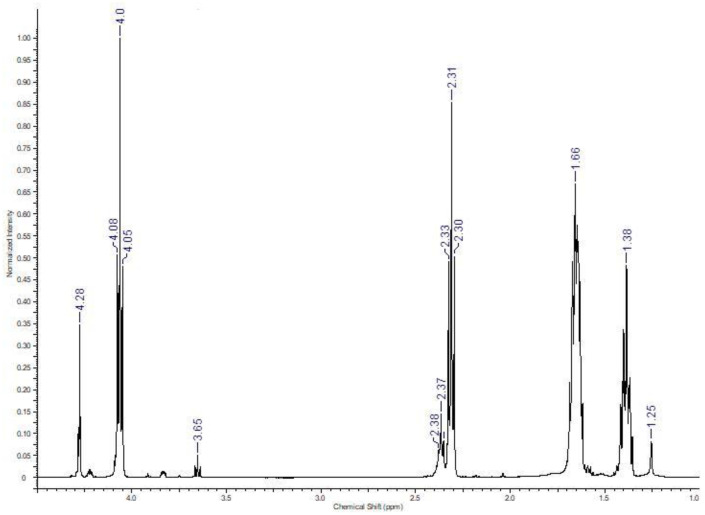
^1^H NMR spectrum of PCL_5000_-PEA_2500_-PCL_5000_ sample in CDCl_3_.

**Figure 6:**
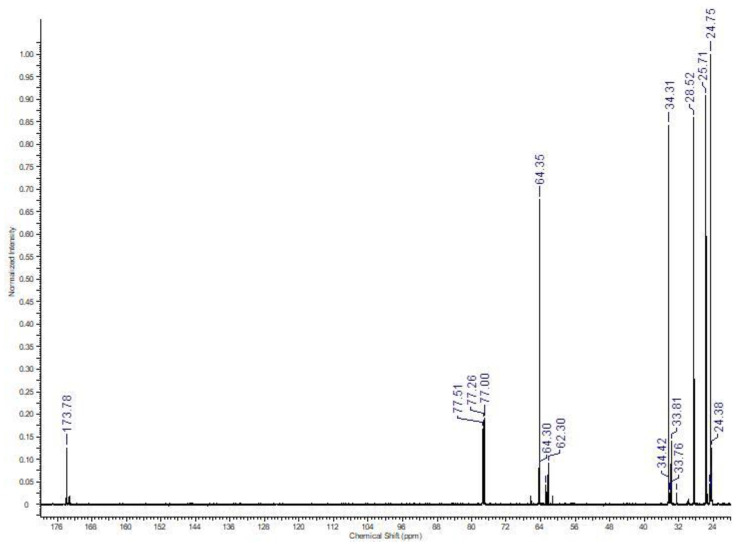
^13^C NMR spectrum of PCL_5000_-PEA_2500_-PCL_5000_ sample in CDCl_3_.

**Figure 7:**
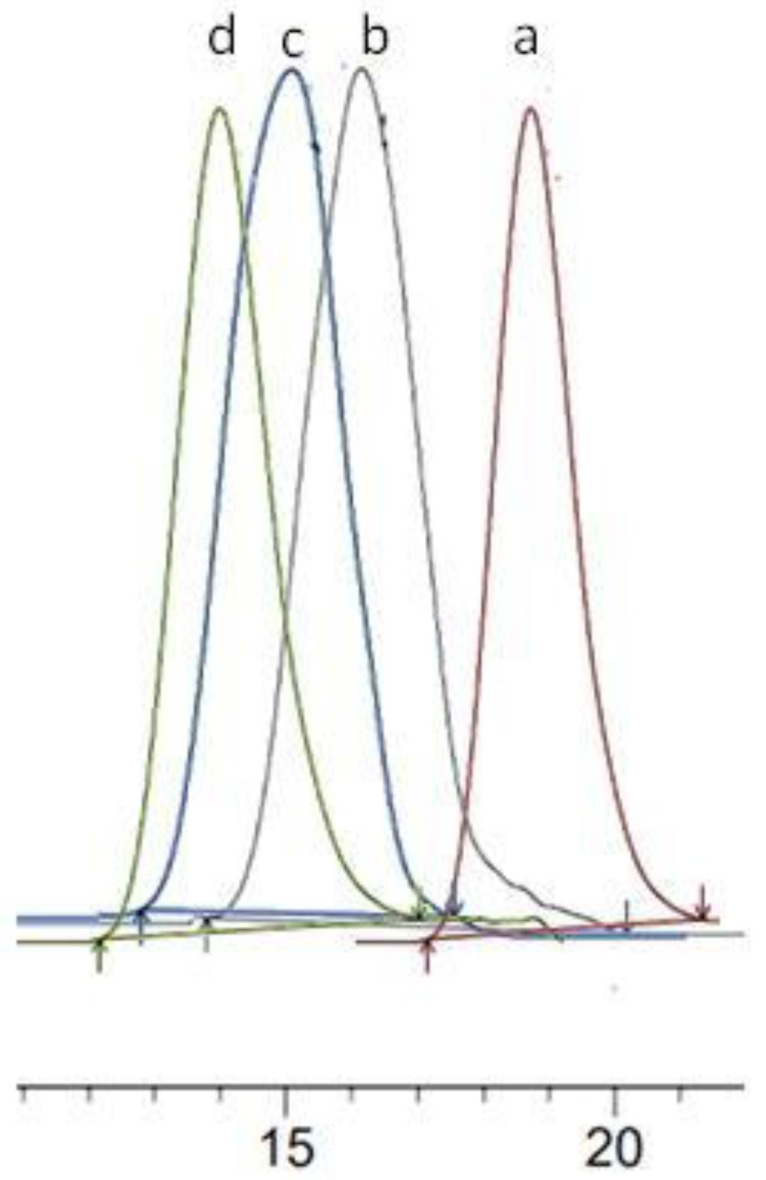
Superposition of SEC chromatograms of PEA-2 (a), PCL_2000_-PEA_2500_-PCL_2000_ (b), PCL_5000_-PEA_2500_-PCL_5000_ (c) and PCL_7000_-PEA_2500_-PCL_7000_ (d).

A typical ^1^H NMR spectra of the sample PCL_5000_-PEA_2500_-PCL_5000_ is provided in Figure 5, whereas Figure 6 shows the ^13^C NMR spectra for the same sample. In Figure 7, the SEC chromatograms for different copolymer samples are illustrated.

In the ^1^H-NMR spectrum of the copolymer, not only the specific signals of PEA can be found, but also the characteristic peaks of the PCL. The peak at 4.06 ppm corresponds to the CH_2_-OH, at 2.31 ppm to the CH_2_-C=O, whereas those at 1.25, 1.38, and 1.66 ppm to the other CH_2_ groups of the PCL chains. A similar conclusion can be drawn from the ^13^C NMR spectrum of the PCL_5000_-PEA_2500_-PCL_5000_ sample.

Figure 7 shows typical monomodal SEC curves of different copolymer samples which indicate the successful synthesis of these copolymers.

The molecular characteristics of all the copolymer samples obtained in this study are summarized in Table 3.

From Table 3 it appears that a homologues series of PCL-b-PEA-b-PCL copolymers were obtained starting from the dihydroxy-PEA. Then, it seems that the M_n_ values calculated by ^1^H NMR are close to the theoretical values whereas the dispersity Đ values are smaller than two, which is characteristic of the polymerization method used in this study. Concerning the thermal properties of the obtained copolymers, it can be observed that T_m_, T_g,_ and T_d_ increase with the increase in the PCL sequence length, as was expected. At low PCL sequence lengths, for the samples PCL_1000_-PEA_2500_-PCL_1000_ and PCL_2000_-PEA_2500_-PCL_2000_, the thermal properties of the copolymers seem to be lower than that of the commercial PCL homopolymer having an M_n_ of 10,000 g/mol [32]. Nevertheless, by increasing the M_n_ of the PCL blocks, the values of T_m_, T_g,_ and T_d_ were improved.

### 3.3. Degradation Behavior of PCL-b-PEA-b-PCL Block Copolymers

**Figure 8:**
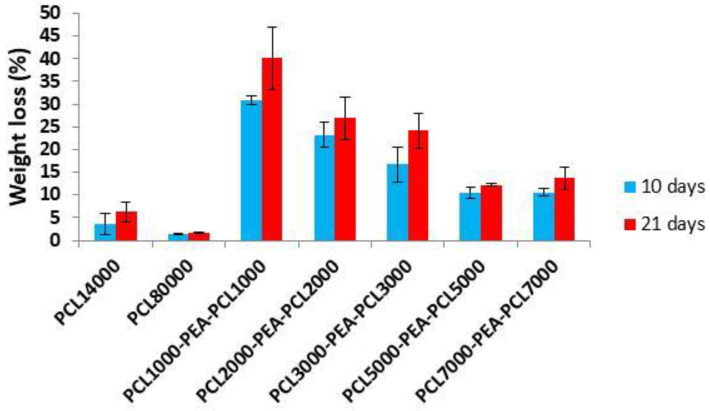
Weight loss profiles of PCL homopolymers and PCL-b-PEA-b-PCL copolymers.

Weight loss profiles as a function of incubation time (Figure 8) revealed the degradation ability of investigated PCL-b-PEA-b-PCL copolymers and of the PCL homopolymers of different molecular weights (10,000 and 80,000 g/mol) that were submitted to degradation for the comparison purpose. Enzymatic hydrolysis was performed in phosphate buffer solution (pH 7.4, 37 °C over 10 and 21 days) in which bacterial cell-free extract containing enzymes *Pseudomonas lipase* PAO1 was added. Numerous in vitro studies revealed that different types of enzymes appeared as highly active toward polymers containing hydrolyzable ester bonds in their chains, from esterase [33] and cutinase [34] to lipase-type enzymes as preferentially used for PCL-based polymers. Lipase-type enzymes are known as highly active and capable to break the ester bonds in PCL-based polymers as a hydrophobic substrate [35]. The use of bacterial cell-free extract instead of pure commercially available enzymes was introduced for the first time on PCL-PEO block copolymers [36] and, in the presented study, the *Pseudomonas lipase* PAO1 was applied for the first time on PCL-b-PEA-b-PCL copolymers. This degradation test (cell-free extract containing *Pseudomonas lipase*) gave an insight into the biodegradation properties for a relatively short period of time and correlate copolymers’ structure and composition to their susceptibility to specific enzyme attacks. According to the obtained results, PCL-b-PEA-b-PCL copolymers are highly susceptible to enzymatic hydrolysis under tested conditions in comparison to PCL homopolymers that showed small weight loss even after 21 days of investigation due to the resistance to enzymes activity over the tested period (PCL10000 about 6 wt.%, while PCL80000 less than 4 wt.%). Copolymer molecular weight appeared as the most affecting factor that dictates degradation ability. Therefore, PCL_1000_-PEA_2500_-PCL_1000_, a copolymer with the smallest molecular weight in the series, lost about 40% of its mass after 21 days. The extension of PCL block length, which means the higher molecular weight of copolymers, resulted in reduced weight loss, and the copolymers PCL_2000_-PEA_2500_-PCL_2000_ and PCL_3000_-PEA_2500_-PCL_3000_ indicated weight loss of about 26 wt.% and 24 wt.%, respectively. Copolymers PCL_5000_-PEA_2500_-PCL_5000_ and PCL_7000_-PEA_2500_-PCL_7000_ exhibited similar weight losses after 10 and 21 days pointing out that enzymatic degradation rate had no correlation with molecular weight when these values are higher than 4000 g/mol per PCL block, which is in agreement with some previous reports [34,37]. However, copolymer PCL_7000_-PEA_2500_-PCL_7000_ with a weight loss of ~11 wt.% after 10 days of incubation had about 50% higher loss in comparison to its commercial homopolymer analogue PCL10000 (weight loss of 6 wt.%) indicating that introduction of a PEA block into PCL chains improved its biodegradability.

#### FTIR Analysis of Degraded Samples

**Figure 9:**
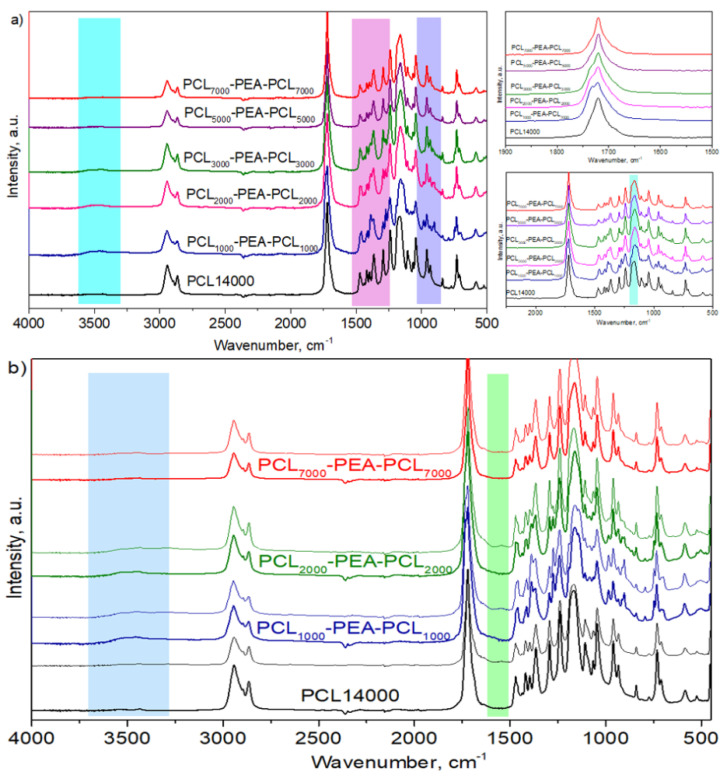
FTIR analysis of (**a**) control/blank polymer samples and (**b**) representative spectra of PCL homopolymer and PCL-b-PEA-b-PCL copolymers after 21 days of degradation in the presence of *Pseudomonas lipase* PAO1 enzymes.

Fourier transform infrared spectroscopy (FTIR) is a useful tool to estimate the changes in the polymer structure after enzymatic degradation. In Figure 9a, the FTIR spectrum of all started polymer samples was shown. All PCL characteristic peaks were detected in copolymer samples associated with PEA characteristic peaks. While in PCL homopolymer, carbonyl group vibrations (−C=O) appeared as a sharp peak at 1720 cm^−1^, the corresponding band in PCL-b-PEA-b-PCL copolymers was broader and split into two peaks (more like the shoulder of −C=O peak from PCL) located at 1720 and 1733 cm^−1^, due to the presence of PEA blocks. When the PCL blocks’ lengths were 5000 g/mol and longer, this peak splitting was hardly visible and the band looked similar to these in PCL homopolymer. The C-C and C-O stretching vibrations in the crystalline phase of PCL homopolymer were detected at 1294 cm^−1^, while in the PCL-b-PEA-b-PCL copolymer structure, a new peak at 1272 cm^−1^ coming from the same groups but in PEA blocks introduced into PCL chains, was detected. The peak coming from the C-C and C-O stretching modes in the amorphous phase of PCL was located at 1164 cm^−1^, and the appearance of this peak changed and became broader in the PCL-b-PEA-b-PCL copolymers. The hydroxyl functional groups peak, in the area of 3000 to 3500 cm^−1^, was observed in the structure of PCL-b-PEA-b-PCL copolymers with molecular weights lower than 10,000 g/mol, i.e., in the copolymers with PCL chains length of 3000 g/mol and lower, where it was possible to detect the free −OH end groups due to the short PCL blocks length. Further, in all recorder spectrums, −CH_2_ vibration bands located in the area of 2943 cm^−1^ and 2870 cm^−1^, as well as in the area of 708–730 cm^−1^, could be seen.

In Figure 9b, representative spectra highlighted the structural changes of the PCL-b-PEA-b-PCL copolymers that suffered from the highest weight loss in the series of tested copolymers.

The structure of PCL homopolymer (PCL14000) with a negligible weight loss after immersion in phosphate buffer containing *Pseudomonas lipase* PAO1 remained unchanged since the absorption peaks of degraded samples were quite similar to the initial ones. According to the spectra of PCL_1000_-PEA_2500_-PCL_1000_ and PCL_2000_-PEA_2500_-PCL_2000_ copolymers with remarkable weight loss, the hydroxyl group characteristic peak became broader (3000–3500 cm^−1^), but also a new peak at the wavenumber of 1560 cm^−1^ appeared. Broadening of hydroxyl group characteristic peak could be attributed to the degradation products with carboxyl and hydroxyl end groups after the hydrolysis of ester bonds in copolymer chains [38]. An additional peak in degraded samples might be related to the stretching of –COO^−^ groups of carboxylic acids that were formed after enzymatic degradation [39].

## 4. Conclusions

A homologues series of polyester-based copolymers were obtained in the present study. The starting point was a dihydroxy-PEA precursor and PCL-b-PEA-b-PCL samples were synthesized by the mass polycondensation of the ε-CL at high temperatures. The obtained M_n_ of the PEA was around 2500 g/mol whereas the M_n_ of the PCL blocks varied between 1000 and 10,000 g/mol. According to the performed physicochemical analysis, the molecular characteristics of the copolymers are directly correlated with the amount of the PCL sequences. Enzymatic degradability studies, as a function of time, showed that the presence of the PEA sequence increases the degradability rate of the copolymers in comparison to the commercial PCL homopolymers. The present study proves that the degradability of these copolymers, which is an essential characteristic for biomedical applications, can be modulated as a function of the ratio between the PEA and PCL blocks. Therefore, it can be admitted that these copolymers can be used for the preparation of biodegradable and biocompatible drug delivery systems which will be investigated in a further study.

## Figures and Tables

**Table 1:** Experimental conditions and molecular characteristics of the obtained di-hydroxylated polyesters.

Parameters	PEA-1	PEA-2	PEA-3 ^a^
Duration ^b^ (h)	0.5	3	3
Pression (mbar)	0.026	0.026	0.026

^a^ The synthesis was carried out in the absence of the catalyst; ^b^ the duration of stage three.

**Table 2:** Molecular characteristics of the obtained di-hydroxylated polyesters.

Molecular Characteristics	PEA-1	PEA-2	PEA-3
M_n_ ^a^ (g/mol)	960	2480	670
M_n_ ^b^ (g/mol)	1045	2420	670
M_n_ ^c^ (g/mol)	1415	3275	1000
T_m_ ^d^ (°C)	43.0	45.5	42.0
T_g_ ^e^ (°C)	−52	−47.5	−52.5
T_d_ ^f^ (°C)	278.5	316.0	242.0

^a^ determined by ^1^H NMR; ^b^ determined by ^13^C NMR; ^c^ determined by SEC using PS standards; ^d^ melting temperature determined by DSC; ^e^ glass transition temperature determined by DSC; ^f^ degradation temperature determined by TGA and corresponds to a mass loss of 5%.

**Table 3:** Molecular characteristics of PCL-b-PEA-b-PCL copolymers.

Samples	M_n(NMR)_ PEA (g/mol)	M_nth_ PCL (g/mol)	M_n(NMR)_ PCL (g/mol)	M_n(SEC)_ Cop (g/mol)	Đ cop	T_m_^d^ Cop (°C)	T_g_^e^ Cop (°C)	T_d_^f^ Cop (°C)
PCL_1000_-PEA_2500_-PCL_1000_	2480	2000	2900	5500	1.73	46.5	−48.5	320.3
PCL_2000_-PEA_2500_-PCL_2000_		4000	4800	7200	1.99	58.8	−52.1	325.2
PCL_3000_-PEA_2500_-PCL_3000_		6000	6800	9400	1.78	61.2	−55.7	348.9
PCL_5000_-PEA_2500_-PCL_5000_		10,000	10,700	14,100	1.65	63.5	−57.3	360.4
PCL_7000_-PEA_2500_-PCL_7000_		14,000	15,100	18,300	1.87	65.2	−58.5	375.6
PCL_10000_-PEA_2500_-PCL_10000_		20,000	21,500	23,200	1.98	67.9	−61.2	398.5

## Data Availability

The data presented in this study are available on request from the corresponding author.
